# Secondary use of health care data and left-over biosamples within the ‘Medical Informatics Initiative’ (MII): a quasi-randomized controlled evaluation of patient perceptions and preferences regarding the consent process

**DOI:** 10.1186/s12911-022-01922-6

**Published:** 2022-07-15

**Authors:** Sybille Roschka, Torsten Leddig, Mandy Bullerjahn, Gesine Richter, Wenke Liedtke, Martin Langanke, Wolfgang Hoffmann

**Affiliations:** 1 Institute for Community Medicine, Section Epidemiology of Health Care and Community Health, University Medicine Greifswald, Ellernholzstr. 1-2, 17487 Greifswald, Germany; 2 Core Unit Data Integration Center, University Medicine Greifswald, Walther-Rathenau-Straße 49a, 17489 Greifswald, Germany; 3 Division Patient Management, Department Patient Admission, University Medicine Greifswald, Fleischmannstr. 8, 17489 Greifswald, Germany; 4 Institute of Experimental Medicine, Division of Biomedical Ethics, Kiel University, University Hospital Schleswig-Holstein, Kiel, Germany; 5 Department of Social Work, Protestant University of Applied Sciences Rhineland-Westphalia-Lippe, Immanuel-Kant-Str. 18-20, 44803 Bochum, Germany

**Keywords:** Broad consent, Consent process, Consent implementation, Patient preferences, ‘Medical Informatics Initiative’

## Abstract

**Background:**

Data collected during routine health care and ensuing analytical results bear the potential to provide valuable information to improve the overall health care of patients. However, little is known about how patients prefer to be informed about the possible usage of their routine data and/or biosamples for research purposes before reaching a consent decision. Specifically, we investigated the setting, the timing and the responsible staff for the information and consent process.

**Methods:**

We performed a quasi-randomized controlled trial and compared the method by which patients were informed either in the patient admission area following patient admission by the same staff member (Group A) or in a separate room by another staff member (Group B). The consent decision was hypothetical in nature. Additionally, we evaluated if there was the need for additional time after the information session and before taking the consent decision. Data were collected during a structured interview based on questionnaires where participants reflected on the information and consent process they went through.

**Results:**

Questionnaire data were obtained from 157 participants in Group A and 106 participants in Group B. Overall, participants in both groups were satisfied with their experienced process and with the way information was provided. They reported that their (hypothetical) consent decision was freely made. Approximately half of the interested participants in Group B did not show up in the separate room, while all interested participants in Group A could be informed about the secondary use of their routine data and left-over samples. No participants, except for one in Group B, wanted to take extra time for their consent decision. The hypothetical consent rate for both routine data and left-over samples was very high in both groups.

**Conclusions:**

The willingness to support medical research by allowing the use of routine data and left-over samples seems to be widespread among patients. Information concerning this secondary data use may be given by trained administrative staff immediately following patient admission. Patients mainly prefer making a consent decision directly after information is provided and discussed. Furthermore, less patients are informed when the process is organized in a separate room.

**Supplementary Information:**

The online version contains supplementary material available at 10.1186/s12911-022-01922-6.

## Background

An increasing amount of routine health-care data is stored in electronical formats such as electronic medical records. The utilization of this type of data for medical research can enrich the scientific knowledge based on (clinical) studies, and it has the potential to further improve the prevention, diagnosis, prediction and the treatment of diseases. Such ‘real world data’ and hence ‘real world evidence’ [1] offers several advantages. Because ‘real world data’ are observational in nature, they help understand how health interventions work within routine care. Furthermore, in contrast to different study types, large numbers of patients, including those with multimorbidity, can be considered for analysis, thus the analysis of such data may help to gain a better knowledge base for specific patient groups. The German Medical Informatics Initiative (MII) [2] is a nationwide project funded by the German Federal Ministry of Education and Research (BMBF) with the goal of digitally connecting University Hospitals so that data from routine health-care and (clinical) trial data can be exchanged and used for treatment and research purposes [3]. The project is of great importance to the digitisation of medicine and the networking of University Hospitals in Germany by using innovative data architectures and software solutions. In the MII, the University Medicine Greifswald (UMG) provides tools for establishing General Data Protection Regulation (GDPR)-compliant trust centre solutions [4], implementing record linkage [5], pseudonymization [4], and for consent management [6, 7].

Prerequisite for a secondary use of clinical data in research is the informed consent of the patient. In the context of the MII, clinical data and left-over biosamples (biospecimen, e.g. blood, urine or cells) are collected over a period of 5 years after consent is given. These data and biosamples can be stored for a duration of 30 years. Since most future projects and research questions are not foreseeable at the moment of consent, a ‘broad consent’ [8–10] is applied. The MII broad consent is restricted to medical research only, but not limited to specific projects or diseases [11, 12].

To facilitate the obtainment of written consent, harmonised patient information and consent forms (standardised modularized template forms) [13] have been developed to be used across the MII member sites. In addition to these forms, the MII requires the implementation of a verbal explanation, which should be followed by questions and answers between the patient and staff, so that the patient obtains all pertinent information to make an informed consent. It is recommended to present issues and documents in an intelligible and short manner, to use plain language, and to additionally have a one-on-one meeting with the patient in order to help the patient comprehend, since the information presented is complex, technical, and specialized [14–17].

For an implementation of the MII broad consent, concrete, suitable and ethically sound processes for information and consent need to be developed, to ensure an independent and informed patient decision. Little is known about how these processes can best be organized. What are the needs of patients for the organisation of this information and consent process? Where, when and by whom—considering site specific circumstances—can the process best be applied? Is the moment of patient admission and the admission session adequate for the process? How much time do patients need to consider whether or not to provide consent? These are example questions that need to be investigated.

The purpose of this study was to examine how in-patients (representing the main group of patients within the hospital) perceive two possible organizational settings regarding the consent process for the scientific use of their routine health-care data and left-over samples, and to explore the needs and preferences that patients articulated. With our analysis, we contribute to the development of an ethically sound and feasible process for MII consent implementation within daily routine at a University Hospital.

## Methods

This study was performed as a quasi-randomized cross-sectional trial in the context of a University Hospital in Germany.

Ethical approval was obtained by the local Research Ethics Committee (Reg. No. BB 082/19).

The sample comprised consecutive patients. Patients were included if they were ≥ 18 years with admission to the University Medicine Greifswald. They were excluded if they fulfilled at least one of the following exclusion criteria:

- Emergency hospitalisation
- Outpatient admission
- Participation in this study in the context of a previous admission
- Insufficient knowledge of the German language.

Where necessary, participants with cognitive disabilities were assisted by their (legally authorized) representative. The representative acts in the (objective) interest of the patient and respects his/her (presumed) will.

Two alternative schemes of patient information and obtaining broad consent processes were compared:

- Group A: The information and consent process (MII consent) was performed in the patient admission area subsequent to completion of patient admission by the same staff member.
- Group B: The information and consent process (MII consent) was performed after patient admission in a separate room (approximately 70 m apart) by a different staff member.

The participants were allocated to Group A or B by a quasi-chance process determined by organizational matters (e.g. staff service schedule, number of patients waiting for admission) independent from study issues.

The need for additional time—for example to carefully read the patient information or to reflect on the information obtained—to decide for or against provision of consent was evaluated in both groups.

### Procedure

A leaflet and the MII patient information were laid out in the waiting area of the patient admission area for self-study purposes. Upon completion of hospital admission, patients and their (legal) representatives (if present) were provided with information about the study. Consent to participate in this study was given verbally. According to group allocation, participants either remained sitting to take part in the study (Group A) or were asked to change the room (Group B) to proceed. Participation in the study comprised two parts:

1. The MII broad consent process, where participants were informed about the scientific use of routine data and left-over samples. The participants knew that this process was simulated; no true consent was obtained within this study.
2. An interview with questions on how the respective MII information and consent process was perceived, using a questionnaire specifically developed for this study (see Additional files 1, 2, 3, and 4).

During the MII consent process (part 1), participants were informed in a structured way in accordance with the MII patient information (v. 1.6a) by specifically trained administrative staff. Topics included the collection of routine patient data and left-over biosamples for research purposes within the following 5 years, data storage for 30 years, information on data safety and possible informational risks. After all open questions were answered, participants were asked whether they already had (hypothetically) decided for or against provision of their consent at this time-point or rather needed some additional time for their decision-making process, for example to read the written information, to rethink and discuss the scientific use of data and left-over samples. This led to one of the following options for the subsequent process:

- Hypothetical decision for or against the scientific use of the individual routine data and/or left-over samples directly after information provision without further time for consideration.
- Usage of additional time for consideration before hypothetically deciding for or against the scientific use of the individual routine data and/or left-over samples.
  This option involved either:
  A visit of a study staff member at an appointed later point of time on the ward (b1), or
  A written response by the participant (b2).

After the participants took their decision on the following process a, b1 or b2, they were asked about their preferences for and experiences with the MII consent process (part 2). Those participants choosing time for consideration (process b1 or b2) were provided with written material (MII patient information and leaflet). With the participants choosing process b1, an appointment for a second study visit on the ward was made. This additional visit was intended to offer the possibility to ask further questions (part 1) and to perform a questionnaire-based interview (part 2). Those participants choosing process b2 received a short questionnaire to fill-in and to hand it over to the staff on the ward.

Overall, we developed four different versions of our anonymous questionnaire. Each one was adapted to the respective process and context: (1) patient admission without need for additional time, or (2) patient admission with additional time needed, or (3) separate room without need for additional time, or (4) separate room with additional time needed. All questionnaires comprised Likert-scale items (0/25/50/75/100) as well as nominal items. The questionnaires addressed the topics consent decision, free decision-making, setting, staff organisation, time for consideration between information session and consent, information sources and demographic information. In addition to this information provided by the participants, the study staff was asked to rate the participants’ individual understanding of the MII consent, the perceived independence of patient admission and consent process and whether the consent process caused any stress or strain to participants. Study staff also documented questions asked by the participants as well as some general information, such as day of admission and time needed for information provision and consent decision.

The questionnaire data were analysed descriptively using the Statistical Package for the Social Sciences (SPSS Statistics for Windows, Version 26 (IBM Corp., Armonk, N.Y., USA)).

The study was preceded by a pilot study with 22 participants. It was used to improve the understandability of the questionnaires and the communicative strategies of study staff members during consent information.

## Results

Participants were recruited over a period of 12 weeks. A total of 915 patients (Group A: n = 380; Group B: n = 535) were informed about the possibility to participate in the present study after their hospital admission was completed. Of those, 368 showed an interest (Group A: n = 157; Group B: n = 211). All 157 interested individuals in Group A were then informed about the study’s background, aims, procedures and data processing, and performed the study procedures, i.e. the MII consent process (part 1) and the questionnaire-based interview (part 2). Of Group B, 106 individuals showed up in the separate room and participated in the study procedures. They were informed the same way as group A participants. Four additional individuals declined to perform the study procedures (at this time-point) after they arrived in the separate room. The remaining 101 individuals did not show up in the separate room.

The main characteristics of individuals taking part in the study procedures (part A and B) are described in Table 1.

**Table 1:** Characteristics of participants performing the study procedures

	Group A	Group B	Groups combined
*Age, years (n* = *263)*			
18–27	5 (3.2%)	1 (0.9%)	6 (2.3%)
28–37	12 (7.6%)	6 (5.7%)	18 (6.8%)
38–47	27 (17.2%)	12 (11.3%)	39 (14.8%)
48–57	34 (21.7%)	25 (23.6%)	59 (22.4%)
58–67	44 (28.0%)	39 (36.8%)	83 (31.6%)
68–77	27 (17.2%)	18 (17.0%)	45 (17.1%)
78–87	8 (5.1%)	4 (3.8%)	12 (4.6%)
88 +	0 (0.0%)	1 (0.9%)	1 (0.4%)
*Sex (n* = *263)*			
(female/male)	72 (45.9%)/85 (54.1%)	53 (50.0%)/53 (50.0%)	125 (47.5%)/138 (52.5%)
*Legal representative present (n* = *261)*			
(yes/no)	2 (1.3%)/154 (98.7%)	1 (1.0%)/104 (99.0%)	3 (1.1%)/258 (98.9%)
≥ *1 Previous admission(s) (n* = *263)*			
(yes/no)	113 (72.0%)/44 (28.0%)	63 (59.4%)/43 (40.6%)	176 (66.9%)/87 (33.1%)

The individual consent process duration ranged between five and eight minutes. Questions regarding the MII consent were asked in 26 cases (9.9%) (Group A: n = 8, 5.1%; Group B: n = 18, 17.0%).

One participant (Group B) chose the opportunity to take extra time for consideration (opting for a written participant response; process b2) before hypothetically deciding for or against the scientific use of the individual routine data and/or left-over samples. However, we did not receive the filled-in short questionnaire from this participant. All other participants (n = 262) preferred to take the hypothetical decision directly after the information session without additional time to consider.

Overall, the (hypothetical) willingness to provide a broad consent (MII consent) was very high, all participants (n = 157, 100%) in Group A and 103 of 105 (98.1%) in Group B stated that both their routine data and left-over samples may be used for medical research.

When participants reflected on the information session itself, all of them stated that the verbal information was helpful (n = 263; 100%) and that they did not want to speak with a physician in addition to the provided information session (n = 263; 100%). They largely felt well informed (Table 2, Q4).

**Table 2:** Participant perceptions regarding free consent decision (Q1, Q2, Q3) and sufficient information (Q4)

Q1. Did you feel to be able to freely decide for or against the scientific use of patient data and left-over samples? (0/25/50/75/100; 0 = No free decision; 100 = Free decision) (n = 262)

Q2. Are you worried of being disadvantaged in case you decide against the scientific use of your patient data and left-over samples? (0/25/50/75/100; 0 = No concerns, 100 = Max. concerns (n = 262)

Q3. Was it clear to you, that both conversations—for patient admission and to inform about the scientific use of patient data and left-over samples—are completely independent from each other? (0/25/50/75/100; 0 = Not clear at all, 100 = Perfectly clear) (n = 263)

Q4. At this time-point, do you feel being sufficiently informed about the scientific use of patient data and left-over samples? (0/25/50/75/100; 0 = Absolutely insufficiently informed, 100 = Max. informed) (n = 262)

We laid out for all patients the written MII patient information and a leaflet in the waiting area. Only few participants went through the documents: Nine participants (3.4%) (Group A: n = 5, 3.2%; Group B: n = 4, 3.8%) had skimmed through the written MII patient information, and no one reported that he or she fully read it. Similarly, only one participant (0.4%) fully read the leaflet (Group B), and five (1.9%) skimmed through it (all Group A). While the study staff specifically pointed to the respective parts in the MII patient information document during patient information, it was not intended for the participants to (re)read the whole document meanwhile. However, only 2.7% (n = 7) (Group A: n = 2, 1.3%; Group B: n = 5, 4.7%) said that they wanted more time to read the written information. On the other side, two (0.8%) participants (both Group B) said that the written information material would have been sufficient for them (without verbal information). One-fifth of participants (n = 57; 21.7%) (Group A: n = 15, 9.6%; Group B: n = 42, 39.6%) would have liked to inform themselves already at home before hospital admission.

When asked about the suitability to perform the consent information session directly after patient admission, 96.6% (n = 254) agreed to the timing (Group A: n = 155, 98.7%; Group B: n = 99, 93.4%), while very few said that this would not be suitable.

We also collected information about preferences regarding the setting (separate room or patient admission area) for patient information and provision of consent. None of the 157 participants in Group A, who performed the MII consent process in the patient admission area, had preferred it in a separate room. In a similar manner, only three of the 106 participants in Group B (2.8%), performing the process in a separate room, stated that they would have preferred to remain sitting in the patient admission area to be informed about the secondary use of data and left-over samples directly after patient admission. The necessity for the implementation of two meeting rooms to differentiate between patient admission and MII information session was not selected by any of the 157 participants in Group A, while more than half of the participants in Group B (n = 64; 61.5%) stated that two different rooms were necessary to clearly show the independence of the two processes. Performing the information session on the ward in the patient’s room would constitute another setting option. Approximately one-fourth of participants in Group A (n = 45; 28.7%) and somewhat less than half of participants in Group B (n = 44; 41.9%) would consider this option.

The topic “free decision-making” regarding the secondary use of patient data and/or left-over samples was covered by three questions in the questionnaire. Participants’ answers in both groups clearly show a freely made hypothetical consent decision (Table 2; Q1, Q2, Q3).

As an additional assessment, staff rated the perceived participants’ understanding of the information provided and the differentiation between the patient admission process and the MII consent as well as any potential stress or strain caused by the MII consent process. The ratings confirm that the information provided was well understood by most participants from an external point of view, while in few cases, the understanding seemed to be incomplete. Overall, participants in both groups were able to differentiate between admission and MII consent process. Stress or strain caused by the MII consent process played a role for some of the participants (Table 3).

**Table 3:** Staff ratings: understanding of information, differentiation between patient admission and MII consent process and observed stress or strain

Q5. Perceived understanding (0/25/50/75/100; 0 = Did not understand at all, 100 = Fully understood) (n = 263)

Q6. Perceived differentiation between patient intake and MII consent (0/25/50/75/100; 0 = No differentiation, 100 = Confident differentiation) (n = 263)

Q7. Stress or strain caused by MII consent process (0/25/50/75/100; 0 = No stress/no strain at all, 100 = Extreme stress/extreme strain) (n = 263)

## Discussion

In this study, we evaluated patient perceptions and preferences for the MII consent process relating to the secondary use of health care data and/or left-over samples and compared two different processes: (1) the performance of the consent process in the patient admission area directly after patient admission by the same staff member and (2) the performance of this process after patient admission in a separate room by a different staff member.

Almost all results within and between groups were surprisingly homogeneous. It showed that the MII consent process, provided individually by trained administrative staff and comprising five to eight minutes of mainly standardized information with the possibility to ask questions, was overall regarded in both groups as helpful, informative and sufficient. Human contact seems to play an important role for the understanding of study information [14].

Participants in both groups reported that the respective process they had experienced allowed for free decision-making. The timing, i.e. the performance of the process directly subsequent to patient admission, found broad acceptance in both groups. Almost all participants preferred the respective setting they had experienced; those performing the patient information and provision of consent in the patient admission area did not want to perform it in a separate room, and vice versa. Although performing the process in a separate room by different staff may have a potential to reduce possible strain during information provision, it seems to be a challenge for some patients to change the location with potential waiting times. We observed that only approximately 50% of our patients with an interest in study participation did show up in the separate room. It can be expected from this observation that a considerable number of patients would not be informed and offered the opportunity to provide routine data and/or left-over samples for research purposes, if they needed to change the location.

We therefore prefer to organize the consent process in the admission setting directly subsequent to the administrative process and conducted by the same staff member. In either case, patients should be informed in a comprehensible manner of the process of patient admission being completed and that information about the following topic is optional. Our participants in the admission area stated after such an information that they could clearly distinguish between the admission process and the information on the MII consent and that they felt free to take their (hypothetical) consent decision. We conclude that most patients feel comfortable with the information and consent decision in the admission setting.

Although only few patients had a look at the leaflet and the patient information document, we believe it is important to provide as much as information as possible in an understandable manner prior to the oral session. The timing for information provision also plays an important role, since at least one fifth of our participants showed a desire to get the information already at home. Both aspects—the timing and the modes of information—could be part of a new study.

The (hypothetical) consent rate in our study was high. Almost all of our participants stated that they would consent to a scientific re-use of their health-care data and left-over samples. Our findings are in line with other studies which observed that the willingness to support medical research by providing broad consent is generally widespread among patients [17–21].

All but one of our 263 (99.6%) participants decided to take the hypothetical consent decision directly after the consent information and the possibility to ask questions. This very high proportion of participants with no demand for extra time to consider related issues contrasts with the findings by Joffe et al. [22], where only 28% of study participants reported that they had signed to participate in a cancer study at the first meeting. This difference may be explained by the relatively small risks inherent to a secondary use of routine data and/or left-over biosamples and the fact that patients do not need to undergo any additional study procedures (such as experimental treatments, diagnostics or interviews) compared to the potential burdens and risks of a cancer treatment study. However, no real consent was obtained in our study. It can be argued that taking decisions may be faster to some extent, when no apparent consequences are implied.

There are further limitations to this study. Our study gathered experiences of one University Hospital, which may affect generalisability. However, the University Medicine Greifswald is comparable to many other medium sized University Hospitals in Germany. It offers the full spectrum of medical specialties (with the exception of organ transplantation) and all modalities of care. The hospital is located in a city and addresses the general population both in this city and in the adjacent rural area which is typical for other German University Hospitals. Another limitation is the fact that we do not have any information from patients who declined to participate in our study and especially of those 50% in Group B who showed an interest in the study but did not show up in the separate room. While we have no information on any specific reasons of these individuals, we cannot fully exclude that this group of patients systematically differs from the group who performed the study in the separate room. Another limitation could occur from the fact that the questionnaire-based interview and the additional staff assessment could not be performed by an independent staff member due to organisational reasons, so that answers may have been biased. However, this situation was identical for both groups, and participants did not show any signs of discomfort with the situation. Despite these limitations, the study’s clear and consistent pattern of results provides relevant insights into the perceptions, preferences and needs of patients for the broad consent process.

## Conclusions

This study indicates—from a patients’ point of view—that the MII broad consent process, which is aimed to obtain a well-informed consent decision from patients to a secondary use of individual routine health-care data and/or left-over biosamples for medical research purposes, can be organized in the setting of patient admission. Hereby, trained administrative staff can secure both the verbal information and the opportunity to ask questions. There seems to be no difference in acceptance between consent information provision either in the patient admission area with the staff member performing the patient admission or after changing to a separate room with another staff member providing the information. Performing the consent process in a separate room may help to reduce some possible stress and unintentional influence during patient information but seems to cause additional strain probably due to the necessity to reach the separate room and to a possible waiting period. Our results show, that this alternative mode of information and consent would probably cause a large proportion of eligible patients not to be informed about the activity and would effectively exclude these from participation.

Almost all of our participants stated that they may be offered the decision for or against the scientific use of routine data and/or left-over biosamples directly after patient information when all questions are answered. They clearly preferred a fast decision making for this kind of research activity. We anyway recommend to hold a process option available for those patients with a need for extra decision time. In addition, supportive modes of information (e.g. leaflets, posters, instructive movies) may be provided before patients get involved in the verbal patient information and provision of consent.

## Supplementary Information

**Additional file 1.** Questionnaire 1 (Process a: Interview-based questionnaire for participants choosing to take the hypothetical decision for or against the scientific use of the individual routine data and/or left-over samples directly after information provision without further time for consideration.).

**Additional file 2.** Questionnaire 2 (Process b: Interview-based questionnaire for participants choosing additional time for consideration before hypothetically deciding for or against the scientific use of the individual routine data and/or left-over samples.).

**Additional file 3.** Questionnaire 3 (Process b2: For participants choosing additional time for consideration and a written response by the participant.).

**Additional file 4.** Questionnaire 4 (Process b1: For participants choosing additional time for consideration and a visit of a study staff member at an appointed later point of time on the ward.)

## Data Availability

The datasets used and/or analysed during the current study are available from the corresponding author on reasonable request.

## Abbreviations

BMBF: German Federal Ministry of Education and Research
GDPR: General Data Protection Regulation (GDPR)
MII: Medical Informatics Initiative
SPSS: Statistical Package for the Social Sciences
UMG: University Medicine Greifswald
