# Supplementary material for: Secondary use of health care data and left-over biosamples within the ‘Medical Informatics Initiative’ (MII): a quasi-randomized controlled evaluation of patient perceptions and preferences regarding the consent process
Source: BMC Med Inform Decis Mak. 2022 Jul 15;22:184. doi: 10.1186/s12911-022-01922-6 (PMC9287940; doi:10.1186/s12911-022-01922-6)
Supplement: Supplementary file 2 — Additional file 2. Questionnaire 2 (Process b: Interview-based questionnaire for participants choosing additional time for consideration before hypothetically deciding for or against the scientific use of the individual routine data and/or left-over samples.). [file 12911_2022_1922_MOESM2_ESM.docx]

# Questionnaire 2 / Patient perceptions SEction

# - To be filled in by a staff member -

| PFIFF Office Patient Admission Area |
| --- |

| Ward Letterbox |
| --- |

#### What else would you have wished for the information session?

____________________________________________________________________________________________________________________________________________________________________

## free decision making

#### Was it clear to you, that both conversations – for patient admission and to inform about the scientific use of patient data and left-over biosamples – are completely independent from each other?

Not clear at all Perfectly clear

0 25 50 75 100

## Temporal decoupling

#### Had you preferred to inform yourself about the scientific use of patient data and left-over samples at home before hospital admission?

Yes No

#### How do you rate the information session on the scientific use of patient data and left-over samples? *Please rate the individual statements.*

I would have preferred to have had a more detailed conversation. Yes No

The conversation could have been shorter. Yes No

All the necessary information was provided during the information session. Yes No

The written documents alone would have been sufficient for me

(without an information session). Yes No

The personal conversation was helpful. Yes No

All my questions were answered during the conversation. Yes No

I would have liked to talk to a physician in addition. Yes No

I still had questions, but did not ask them. Yes No

It was too much information. Yes No I would have liked more time to read the written information. Yes No

I had time to think about the information provided and could ask all my questions. Yes No

I would have signed anything to finish quickly. Yes No

For me, it goes without saying that I support research at the University Medicine. Yes No

#### From your point of view, is it appropriate to conduct the information session on the scientific use of patient data and left-over samples directly after patient admission?

Yes No

## SPATial DECOUPLING *(PATIENT ADMISSION OR PFIFF VARIANT)*

#### Patient admission area: In your opinion, is the patient admission area suitable for the information session on the scientific use of patient data and left-over samples? *Please rate the individual statements.*

| I found it good to remain seated for a moment after the patient admission and then to be informed about the scientific use of the patient data and left-over samples. | Yes No |
| --- | --- |
| I would have preferred to have the information session on the scientific use of patient data and left-over samples in a separate room. | Yes No |
| It is good that I did not have to change the room for the information session after the patient admission. | Yes No |
| I would have liked a staff member to visit me on the ward for the information session. | Yes No |
| I am happy to put up with an extra walk to a separate room. | Yes No |
| A spatial separation is necessary in order to make the independence of patient admission and information session clear. | Yes No |
| I don't mind where the information session takes place - whether at the counter of the patient admission area or in a separate room or on the ward. | Yes No |

#### PFIFF: In your opinion, is the service office suitable for the information session on the scientific use of patient data and left-over samples? *Please rate the individual statements.*

| I found it good to do the patient admission first and then be free to decide whether I also wanted to be informed about the scientific use of my patient data and left-over samples. | Yes No |
| --- | --- |
| I would rather have stayed at the admission desk to be informed about the scientific use of my patient data and left-over samples right after the patient admission. | Yes No |
| For the information session, a more quiet room than the service office would be more suitable, where you can talk undisturbed. | Yes No |
| I would like a staff member to visit me on the ward for the information session. | Yes No |
| I gladly accepted the additional way into the separate room. | Yes No |
| A spatial separation is necessary in order to make the independence of patient admission and information session clear. | Yes No |
| I don't mind where the information session takes place - whether at the counter of the patient admission area or in a separate room or on the ward. | Yes No |
| The separate room was difficult to find. | Yes No |
| It was physically exhausting for me to get into the separate room. | Yes No |

## Decoupling of staff

#### The discussion for patient admission and the information session on the scientific use of patient data and left-over samples can generally be conducted by one or two different staff members. What is important to you? *Please select the answer that best applies to you.*

For me, *time* is crucial - the fewer different contacts, the faster I get through.

These are all *personal issues* for me that I would prefer to discuss with only one contact person.

For me, the independence of patient admission from involvement in research is crucial. That's

why I think it would be good if two different people would conduct the patient admission and the

information.

For me, the *competence* of the staff is crucial. If I am then better advised, I like to speak with two contacts for the two topics.

For me, it is crucial that the process is *as uncomplicated as possible*. That is why only one (competent) person should conduct both conversations.

I don't mind whether the two conversations are conducted by one or two people.

## Use of information sources

#### Have you … the written patient information in the waiting area

read* skimmed not looked at?

#### Did you … the leaflet ("Brief information") in the waiting area of the patient admission

read⁺ skimmed not looked at?

#### Is the text of the written patient information...?

too short too long just right? No rating possible.

#### *(*Only if the written patient information has been read through beforehand:)* How understandable do you find the patient information?

Not understandable Maximum understandable

0 25 50 75 100

#### *(⁺Only if the brief information (leaflet) has been read through beforehand:)* Is the text of the brief information (leaflet)...?

too short too long just right?

#### *(⁺Only if the brief information (leaflet) has been read through beforehand:)* How understandable do you find the brief information?

Not understandable Maximum understandable

0 25 50 75 100

## QUESTIONS ABOUT THE STUDY PARTICIPANT

#### Information about yourself

Please briefly state your age and gender:

Age (years):

18-27 28-37 38-47 48-57 58-67 68-77 78-87 88+

Gender:

Male Female Diverse

#### Have you previously been admitted as an inpatient at the patient admission area of the University Medicine Greifswald?

Yes.

No, today was the first time.

# Thank you for your cooperation!

# QUESTIONNAIRE 2 / STAFF EVALUATIONS SECTION

# - To be filled in by a staff member -

#### Has the study participant (patient) currently a legally authorized representative?

Yes No

#### Was the patient admitted as an (partially) inpatient or as a preadmission?

inpatient preadmission partially inpatient

#### On which day of the week did the patient admission take place?

Monday Tuesday Wednesday Thursday Friday Weekend

#### Duration of the information session (time of consent to study participation until question about hypothetical consent):

Minutes measured time estimated time

#### Did the patient or representative need to talk about anything other than the study, e.g. questions about the hospital stay, description of the personal situation?

Yes No

#### Did the patient/representative ask questions about the MII consent?

Yes No

#### *(If the patient/representative has asked questions about the MII consent:)*

#### Which questions / topics in particular were addressed?

______________________________________________________________________________________________________________________________________________________________________________________________________________________________________________________
______________________________________________________________________________________________________________________________________________________________________________________________________________________________________________________

#### Did you feel that the patient/representative understood the MII consent?

Did not understand at all Fully understood

0 25 50 75 100

#### Did you feel that the patient/representative was able to differentiate between the patient intake and MII consent conversations?

No differentiation Confident differentiation

0 25 50 75 100

#### Did you have the impression that the conversation about the MII consent was a burden or caused stress for the patient or the representative?

No stress / Extreme stress /

No strain at all Extreme strain

0 25 50 75 100
