# Supplementary material for: Secondary use of health care data and left-over biosamples within the ‘Medical Informatics Initiative’ (MII): a quasi-randomized controlled evaluation of patient perceptions and preferences regarding the consent process
Source: BMC Med Inform Decis Mak. 2022 Jul 15;22:184. doi: 10.1186/s12911-022-01922-6 (PMC9287940; doi:10.1186/s12911-022-01922-6)
Supplement: Supplementary file 3 — Additional file 3. Questionnaire 3 (Process b2: For participants choosing additional time for consideration and a written response by the participant.). [file 12911_2022_1922_MOESM3_ESM.docx]

# Questionnaire 3 / Patient perceptions

# - To be filled in by the participant -

| To be filled in by the staff member: PFIFF Office Patient Admission Area |
| --- |

## Dear study participant,

## thank you for your interest in our study. Following your (pre-/partial) inpatient admission, you were informed by our study staff about the planned scientific use of patient data and left-over biosamples at the University Medicine Greifswald. After the information session, you have requested some time to think about whether you would agree to the use of your data and/or left-over biosamples for research.

## We would be pleased if you would give us feedback using this questionnaire after you have had sufficient time to think about it. The purpose of this questionnaire is to document your perceptions and wishes regarding the information session in a structured manner. Your answers will help us to optimise the planned procedures and the information session.

## **The answering of the questions is voluntary!**

## Your answers will be stored anonymously so that no conclusions can be drawn about your person. Therefore, please do not write your name on the questionnaire.

## You can hand in the completed questionnaire at the patient information desk in the foyer of the University Hospital or on the ward. Please use the enclosed envelope marked "Hauspost".

## If you have received a stamped envelope, it is possible to return the questionnaire through the regular post.

#### Would you now consent to the scientific use of your patient data or leftover biosamples after some time to think about it?

- Scientific use of your patient data

Yes No Decision not possible

- Scientific use of your left-over biosamples

Yes No Decision not possible

#### How did you use the time to consider?

- Have you used the reflection period to let the information "let it sink in"?

Yes No

- Have you used the reflection period to search for information on the topic (for example,

on the internet)?

Yes No

- Have you used the reflection period to speak with relatives/friends?

Yes No

- Did you use the reflection period to move into your room on the ward first?

Yes No

- Have you used the reflection period to try to find answers to unanswered questions?

Yes No

- Have you used the reflection period to read the written patient information?

Yes No Partly

- Have you used the reflection period to read the "Brief information on research with

patient data and left-over biosamples" (leaflet)?

Yes No Partly

#### Do you feel adequately informed about the scientific use of patient data and left-over biosamples at this time?

Absolutely insufficiently Max. informed

informed 0 25 50 75 100

**Did you feel to be able to freely decide for or against the scientific use of patient data and left-over biosamples?**

No free decision Free decision

0 25 50 75 100

**Are you worried of being disadvantaged in case you decide *against* the scientific use of your patient data and left-over samples?**

No concerns Max. concerns

0 25 50 75 100

**How understandable do you find the patient information?**

Not understandable Maximum understandable

0 25 50 75 100

No judgement possible. I have only read / skimmed part of it.

No judgement possible. I did not read it.

**How understandable do you find the „Brief information on research with patient data and left-over biosamples” (leaflet)?**

Not understandable Maximum understandable

0 25 50 75 100

No judgement possible. I have only read / skimmed part of it.

No judgement possible. I did not read it.

#### Information about your person

Please briefly state your age and gender:

Your age:

18-27 28-37 38-47 48-57 58-67 68-77 78-87 88+ years

Your gender:

Male Female Diverse

# THANK YOU FOR YOUR COLLABORATION!
