# Supplementary material for: Secondary use of health care data and left-over biosamples within the ‘Medical Informatics Initiative’ (MII): a quasi-randomized controlled evaluation of patient perceptions and preferences regarding the consent process
Source: BMC Med Inform Decis Mak. 2022 Jul 15;22:184. doi: 10.1186/s12911-022-01922-6 (PMC9287940; doi:10.1186/s12911-022-01922-6)
Supplement: Supplementary file 4 — Additional file 4. Questionnaire 4 (Process b1: For participants choosing additional time for consideration and a visit of a study staff member at an appointed later point of time on the ward.) [file 12911_2022_1922_MOESM4_ESM.docx]

# Questionnaire 4 / Patient perceptions SEction

# - To be filled in by a staff member -

#### After some time to think about the information provided, would you consent to the scientific use of your patient data or left-over biosamples?

- Scientific use of your patient data

Yes No Decision not possible

- Scientific use of your left-over biosamples

Yes No Decision not possible

#### How did you use the time to consider?

- Have you used the reflection period to let the information "let it sink in"?

Yes No

- Have you used the reflection period to search for information on the topic (for example,

on the internet)?

Yes No

- Have you used the reflection period to speak with relatives/friends?

Yes No

- Did you use the reflection period to move into your room on the ward first?

Yes No

- Have you used the reflection period to try to find answers to unanswered questions?

Yes No

- Have you used the reflection period to read the written patient information?

Yes No Partly

- Have you used the reflection period to read the "Brief information on research with

patient data and left-over biosamples" (leaflet)?

Yes No Partly

**Do you feel adequately informed about the scientific use of patient data and left-over biosamples at this time?**

Absolutely insufficiently Max. informed

informed 0 25 50 75 100

**Did you feel to be able to freely decide for or against the scientific use of patient data and left-over biosamples?**

No free decision Free decision

0 25 50 75 100

**Are you worried of being disadvantaged in case you decide *against* the scientific use of your patient data and left-over samples?**

Max. concerns No concerns

100 75 50 25 0

**How understandable do you find the patient information?**

Not understandable Maximum understandable

0 25 50 75 100

No judgement possible. I have only read / skimmed part of it.

No judgement possible. I did not read it.

**How understandable do you find the „Brief information on research with patient data and left-over biosamples” (leaflet)?**

Not understandable Maximum understandable

0 25 50 75 100

No judgement possible. I have only read / skimmed part of it.

No judgement possible. I did not read it.

#### Which information situation applied on the day of your inpatient admission?

The study conversation on the scientific use of patient data and left-over biosamples and the completion of the first questionnaire took place:

Directly at the patient admission

In a separate room („Servicebüro“)

#### Now we would like to ask you for some information about yourself.

Please briefly state your age and gender:

Age (years):

18-27 28-37 38-47 48-57 58-67 68-77 78-87 88+

Gender:

Male Female Diverse

# THANK YOU FOR YOUR COLLABORATION!

# QUESTIONNAIRE 4 / STAFF EVALUATIONS SECTION

# - To be filled in by a staff member -

#### Was the study participant found on the ward at the agreed time?

Yes

No, it took ____ visits to meet the study participant.

No. The study participant was searched for ____x on the ward. He/she could not be met

until the last try.

No. The study participant has already been discharged.

No. The study participant has passed away in the meantime.

No. The study participant was not admitted as an (partial) inpatient.

Other:______________________________________________

#### Duration of the study conversation:

Minutes measured time estimated time

#### Did the patient or representative need to talk about anything other than the study, e.g. questions about the hospital stay, description of the personal situation?

Yes No

#### Did the patient/representative ask questions about the MII consent?

Yes No

#### *(If the patient/representative has asked questions about the MII consent:)*

#### Which questions / topics in particular were addressed?

______________________________________________________________________________________________________________________________________________________________________________________________________________________________________________________
______________________________________________________________________________________________________________________________________________________________________________________________________________________________________________________

#### **Did you feel that the patient/representative understood the MII consent?**

Did not understand at all Fully understood

0 25 50 75 100

#### How do you personally rate the ward visit in terms of added value for the study participant and the cost-benefit ratio?

Little added value for High added value for

the participant the participant 0 25 50 75 100

Little effort for the High effort for the

study staff study staff

0 25 50 75 100
